# Dopamine Signaling Is Essential for Precise Rates of Locomotion by *C. elegans*

**DOI:** 10.1371/journal.pone.0038649

**Published:** 2012-06-13

**Authors:** Daniel T. Omura, Damon A. Clark, Aravinthan D. T. Samuel, H. Robert Horvitz

**Affiliations:** 1 Department of Biology, Massachusetts Institute of Technology, Cambridge, Massachusetts, United States of America; 2 Howard Hughes Medical Institute, Massachusetts Institute of Technology, Cambridge, Massachusetts, United States of America; 3 McGovern Institute for Brain Research, Massachusetts Institute of Technology, Cambridge, Massachusetts, United States of America; 4 Department of Physics and Center for Brain Science, Harvard University, Cambridge, Massachusetts, United States of America; Brown University, United States of America

## Abstract

Dopamine is an important neuromodulator in both vertebrates and invertebrates. We have found that reduced dopamine signaling can cause a distinct abnormality in the behavior of the nematode *C. elegans*, which has only eight dopaminergic neurons. Using an automated particle-tracking system for the analysis of *C. elegans* locomotion, we observed that individual wild-type animals made small adjustments to their speed to maintain constant rates of locomotion. By contrast, individual mutant animals defective in the synthesis of dopamine made larger adjustments to their speeds, resulting in large fluctuations in their rates of locomotion. Mutants defective in dopamine signaling also frequently exhibited both abnormally high and abnormally low average speeds. The ability to make small adjustments to speed was restored to these mutants by treatment with dopamine. These behaviors depended on the D2-like dopamine receptor DOP-3 and the G-protein subunit GOA-1. We suggest that *C. elegans* and other animals, including humans, might share mechanisms by which dopamine restricts motor activity levels and coordinates movement.

## Introduction

Dopamine (DA) signaling plays a key role in many aspects of mammalian nervous system function, including learning, motivation, and motor control [1], [2]. Abnormal DA function has been associated with a variety of disorders, including attention-deficit hyperactivity disorder (ADHD), addiction and Parkinson's disease [1], [3], [4]. Despite advances in the understanding of the genetics and pathophysiology of neurodegeneration in Parkinson's disease and the successful development of several dopamine-based therapies, the molecular mechanisms by which impaired DA signaling leads to movement disorders in Parkinson's disease are poorly understood. The numerous roles of DA and complexity of the human nervous system add to the challenge of identifying a specific role for DA in movement disorders.

The nervous system of the nematode *Caenorhabditis elegans* contains 302 neurons of known connectivity and many evolutionarily conserved neurotransmitters and neuromodulators, including DA [5], [6], [7]. These attributes and the existence of dopamine-deficient mutants make *C. elegans* an attractive organism to use to study the molecular and cellular consequences of impaired DA signaling. *C. elegans* locomotion consists of coordinated dorsal-ventral muscle contractions that propel the nematode through its environment in a sinusoidal fashion [8]. *C. elegans* detects temperature, salts, soluble and volatile chemicals, oxygen, mechanical stimuli and other environmental cues that modulate its motor program [9], [10], [11], [12], [13], [14], [15], [16], [17]. DA in *C. elegans* controls habituation to mechanical stimuli [18], foraging [19] and transitions between crawling and swimming behavior [20]. Mutants lacking DA or the D2-like receptor DOP-3 move faster than wild-type animals on a bacterial lawn, indicating that DA plays a role in slowing *C. elegans* in response to food [16], [21].

In studying the response of dopamine-deficient *C. elegans* mutants to food, we observed that these mutants exhibited highly erratic rates of locomotion. To further characterize this unusual locomotor behavior, we used a video-tracking system that allows the efficient collection of large amounts of high-resolution locomotion data. Our studies revealed a general role for DA in *C. elegans* in restricting the range of average and instantaneous speeds of the animal. This function of DA is behaviorally and genetically distinct from the role of DA in food-dependent slowing.

## Results

### An automated tracking system enables the analysis of new aspects of *C. elegans* locomotor behavior

**Figure 1:**
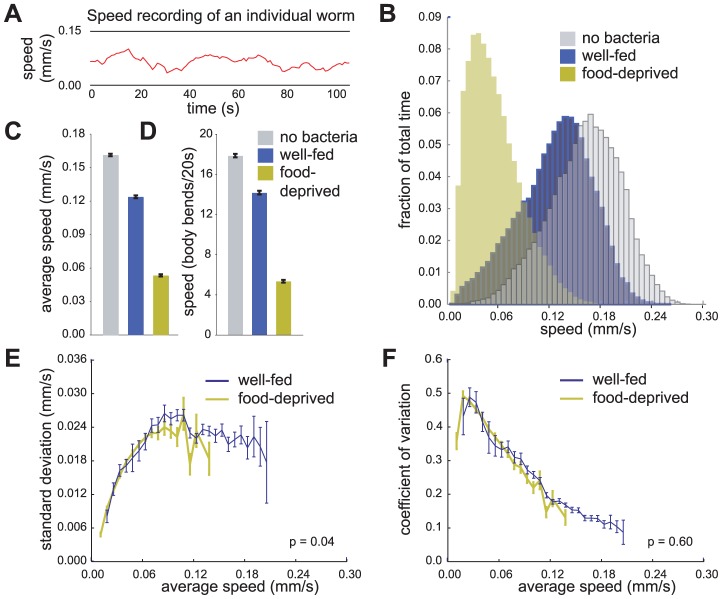
Wild-type *C. elegans* locomotion assayed using an automated locomotion tracking system. (A) A speed recording of an individual worm. (B) Frequency distributions of instantaneous speed recordings of animals in the off-bacteria, well-fed on-bacteria, and food-deprived on-bacteria conditions. Each bin is 0.006 mm/sec wide. (C) Average speeds of animals in each condition. (D) Average speeds of animals as measured by manually counting body bends over 20 sec intervals. Average (E) standard deviations and (F) coefficients of variation of speed measurements within individual tracks for well-fed and food-deprived animals. p-values for differences between well-fed and food-deprived data curves were calculated using two-way ANOVA. Tracks were first grouped by average speed. Each bin is 0.0075 mm/sec wide. Error bars, SEM. (B, C, E, F: n = 1510 off-food, 1174 well-fed, 967 food-deprived animal tracks, mean track length was 53 sec in the automated assay; D: n = 528 off-food, 508 well-fed, 642 food-deprived animals assayed manually).

We used a locomotion tracking system that simultaneously determines the positions of multiple animals on an assay plate at one-second intervals and assembles these position recordings into tracks (Figure 1A) – see materials and methods. Each track contains movement data for an individual animal. By preserving the integrity of each track, this system allows the determination of single-track parameters, such as instantaneous speed, average speed, variance of speed, instantaneous acceleration, duration of accelerations, and magnitude of accelerations. The instantaneous speed recordings of a population of animals can be represented by histograms that show the fraction of time animals move at each speed. These frequency distributions can be overlaid to compare how populations of animals move when exposed to different experimental conditions (Figure 1B).

**Figure 2:**
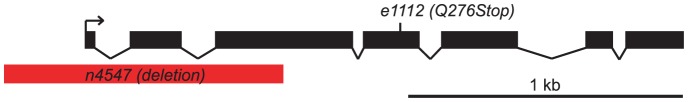
*n4547* is a deletion allele of the tyrosine hydroxylase gene *cat-2.* * n4547* contains a 1007 bp deletion that removes the first, second, and third exons of the *cat-2* gene

**Figure 3:**
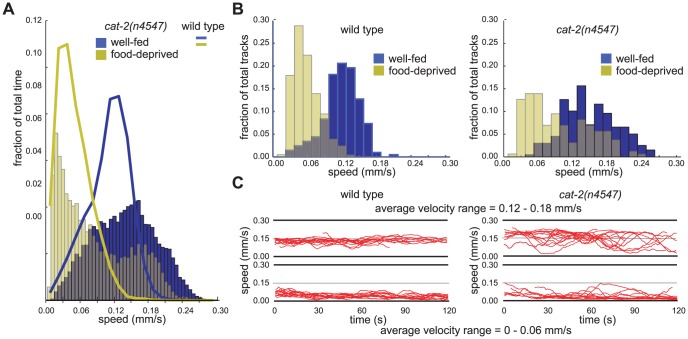
The locomotion rates of *cat-2* mutants were more variable than those of wild-type animals. (A) Frequency distributions of instantaneous speed recordings of *cat-2(n4547)* mutants in each condition. Each bin is 0.006 mm/sec wide. Wild-type frequency distribution outlines are overlaid for comparison. The standard deviations of speed recordings in the well-fed and food-deprived conditions were 0.037 and 0.029 mm/sec for wild-type animals and 0.054 and 0.061 mm/sec for *cat-2* mutants. (B) Frequency distributions of average speeds of individual animals in the well-fed on-bacteria and food-deprived on-bacteria conditions. Each bin is 0.015 mm/sec wide. The standard deviations of average speeds of individual animals in the well-fed and food-deprived conditions were 0.031 and 0.023 mm/sec for wild-type animals and 0.047 and 0.054 mm/sec for *cat-2* mutants. (C) The 15 longest wild-type and *cat-2(n4547)* speed traces with average velocities of 0–0.06 mm/sec and 0.12–0.18 mm/sec overlaid. (A–B: wild-type n = 204 well-fed on-bacteria, 201 food-deprived on-bacteria animal tracks; *cat-2* n = 198, 158, all tracks >20 sec)

**Figure 4:**
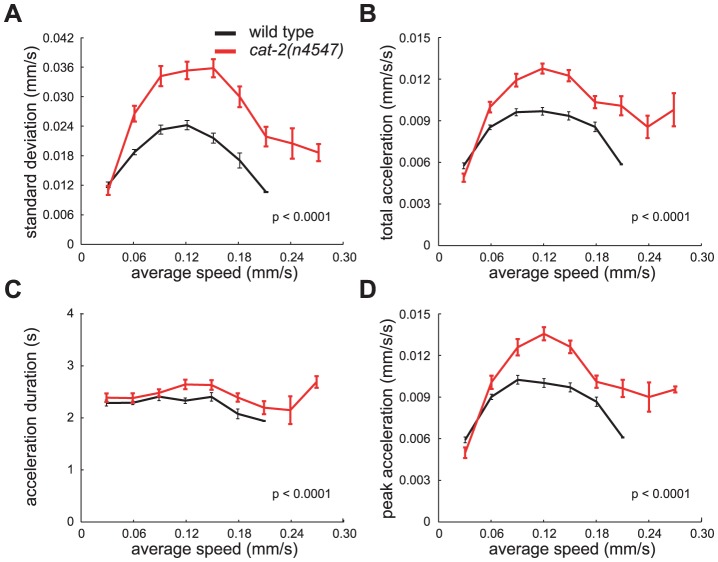
*cat-2* mutants achieve greater peak acceleration, resulting in large fluctuations in the speed within tracks. The average (A) standard deviations of speed measurements within individual tracks, (B) magnitude (root mean square) of accelerations, (C) duration of accelerations, and (D) acceleration peaks for animals on a bacterial lawn. p-values for differences between data curves for each genotype were calculated using two-way ANOVA. Tracks were first grouped by average speed. Each bin is 0.03 mm/sec wide. Error bars, SEMs. (wild-type n = 358 tracks; *cat-2(n4547)* n = 262 tracks, all tracks >30 sec)

**Figure 5:**
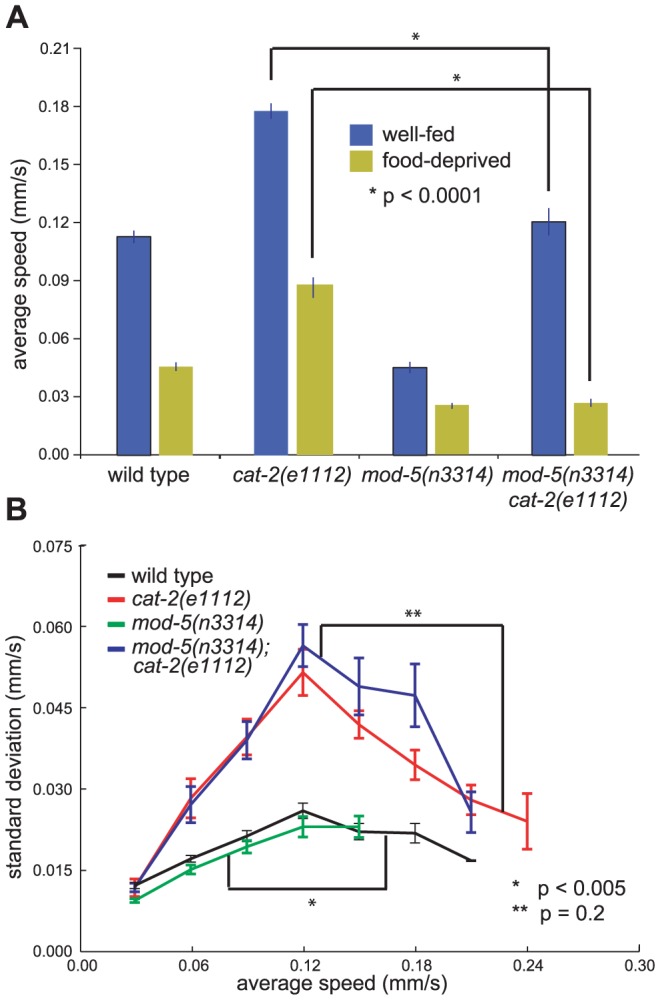
Mutation of *mod-5* suppresses the hyperactivity but not the large speed fluctuations of *cat-2* mutants. (A) Average speeds of wild-type and mutant animals tested in the well-fed on-bacteria and food-deprived on-bacteria conditions. (B) The average standard deviations of speed measurements within individual tracks for animals on a bacterial lawn. p-values for difference in average speeds were calculated using Student's T-test. p-values for differences between standard deviation curves for each genotype were calculated using two-way ANOVA. Tracks were first grouped by average speed. Error bars, SEMs. (A: wild-type n = 120 well-fed, 109 food-deprived tracks; *cat-2(e1112)* n = 123, 96; *mod-5(n3314)* n = 58, 53; *mod-5(n3314) cat-2(e1112)* n = 83, 51; B: wild-type n = 233 tracks; *cat-2(e1112)* n = 131; *mod-5(n3314)* n = 219; *mod-5(n3314) cat-2(e1112)* n = 90, all tracks >30 sec)

**Figure 6:**
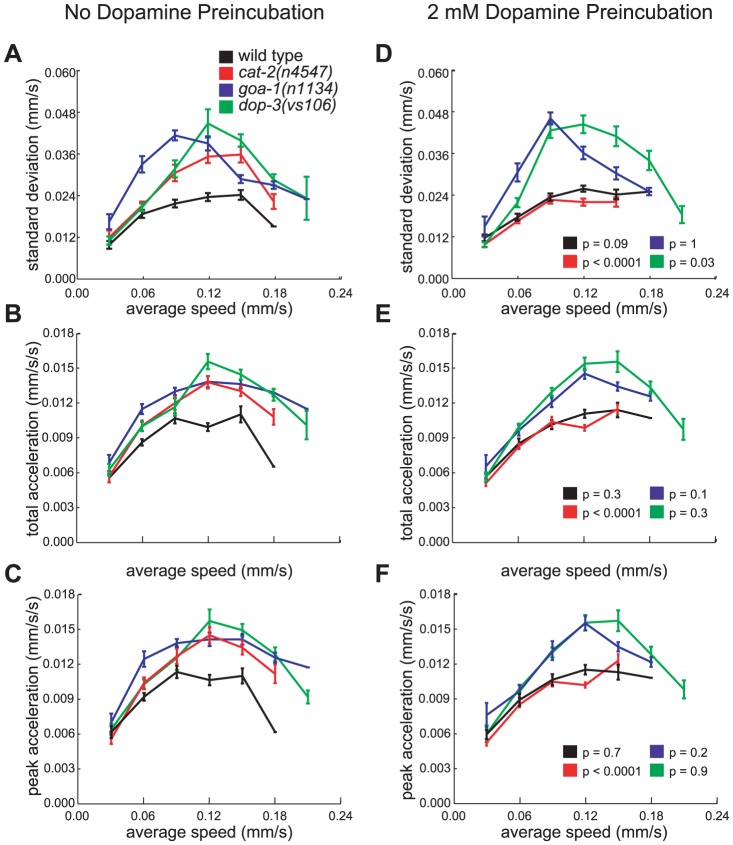
Pre-treatment with exogenous dopamine restores normal locomotion to *cat-2* but not *dop-3* or *goa-1* mutants. The average (A) standard deviations of speed measurements within individual tracks, (B) magnitude (root mean square) of accelerations, and (C) acceleration peaks for animals on a bacterial lawn. The average (D) standard deviations of speed measurements within individual tracks, (E) magnitude (root mean square) of accelerations, and (F) acceleration peaks for DA pre-treated animals on a bacterial lawn. p-values for differences between dopamine-treated and untreated animals of the same genotype were calculated using two-way ANOVA. Tracks were first grouped by average speed. Each bin is 0.03 mm/sec wide. Error bars, SEMs. (wild-type n = 120 no dopamine, 131 DA pretreated animal tracks; *cat-2(n4547)* n = 107, 139; *dop-3(vs106)* n = 122, 128; *goa-1(n1134)* n = 162, 150; all tracks >30 sec)

**Figure 7:**
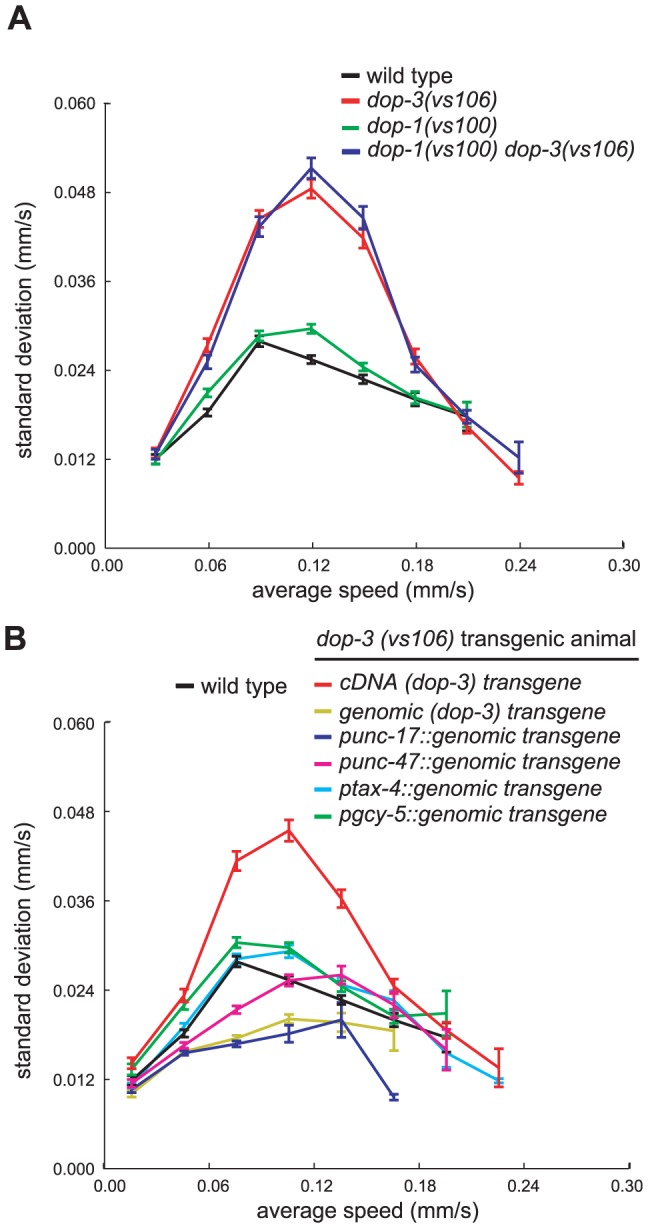
While mutations in *dop-1* fail to suppress *dop-3* speed fluctuations, *dop-3* genomic constructs do, regardless of the promoter used. The average standard deviations of speed measurements within individual tracks for (A) wild-type animals, mutant animals, and (B) transgenic strains on a bacterial lawn. p-values for differences between standard deviation curves for each genotype were calculated using two-way ANOVA. Tracks were first grouped by average speed. Error bars, SEMs. (A: wild-type n = 934 tracks; *dop-3(vs106)* n = 941; *dop-1(vs100)* n = 1045; *dop-1(vs100) dop-3(vs106)* n = 887. B: wild-type n = 934 tracks, *dop-3* cDNA transgenic n = 853, *dop-3* genomic transgenic n = 861, P*_unc-17::dop-3_* genomic transgenic n = 791, P*_unc-47dop-3_* genomic transgenic n = 911, P*_tax-4::dop-3_* genomic transgenic n = 1005, P*_gcy-5::dop-3_* genomic transgenic n = 917. all tracks >30 sec).

To ensure that data collected by our automated tracking system were consistent with our direct observations of *C. elegans* locomotion, we examined the average speed of well fed and food-deprived animals on a bacterial food source as described by Sawin et al. [16]. On a bacterial lawn, well-fed animals moved at 76%, and food deprived animals moved at 32%, of their locomotion rate in the absence of bacteria (Figure 1C). These measurements are comparable to those obtained by manually counting the number of body bends an animal makes during a 20 second interval [16]. Using this manual assay, we observed that on a bacterial lawn well-fed animals moved at 79%, and food-deprived animals moved at 30%, of their locomotion rate in the absence of bacteria (Figure 1D).

### Fluctuations in the speeds of individual animals correlate with average locomotion rates independently of feeding state

We examined the magnitude of fluctuations in the locomotion rates of individual animals by calculating the standard deviations of the intra-track speed measurements. We also calculated the coefficient of variation, which is the standard deviation of intra-track speed measurements divided by the average speed of the animal. The coefficient of variation reflects the amount of intra-track speed variation relative to the average speed of the animal.

Although the standard deviation of intra-track speed measurements changed significantly as the average speed of the animal changed, there was not a statistically significant difference between measurements of well-fed and food-deprived animals. Therefore, fluctuations in speed were not specifically affected by whether the animal had been food-deprived (Figure 1E). As the average speed of animals increased, the instantaneous speed measurements of the animal fluctuated less relative to average speed (Figure 1F). Since the variances of speeds of individual animals correlated with average locomotion rates independently of feeding state, we pooled our data for well-fed and food-deprived animals on bacteria to examine locomotion variability over the largest range of average locomotion rates. Observations of wild-type movement collected on multiple days over many months resulted in nearly identical measurements of intra-track speed fluctuations (data not shown).

### DA signaling is required to restrict the range of speeds at which an individual animal travels

The gene *cat-2* encodes tyrosine hydroxylase [22], which catalyzes the conversion of tyrosine to L-DOPA, the biosynthetic precursor of DA [23]. *cat-2* mutants have greatly reduced levels of DA [7], [22]. We isolated a new allele of *cat-2(n4547)* that contains a large deletion in the *cat-2* open reading frame and presumably represents a null allele of this gene (Figure 2). This allele is easy to identify by PCR and allowed us to backcross and create new strains by directly following the molecular lesion.

Using the automated locomotion tracking system we discovered that the instantaneous speeds of *cat-2* mutants were much more broadly distributed than those of wild-type animals in both the well-fed and food-deprived conditions (Figure 3A). To further define the nature of this abnormality, we examined the average speeds of well-fed and food-deprived individual animals and found that, in addition to *cat-2* mutants being faster [16], the average speeds of individual *cat-2* mutants were also more broadly distributed compared to the average speeds of individual wild-type animals (Figure 3B). We then examined the fluctuations in the speeds of individual animals by comparing the intra-track speed measurements of wild-type and *cat-2* animals. For individual animals moving at the same average speeds, *cat-2* individuals exhibited a greater range of intra-track speeds than did wild-type individuals. Overlaying multiple wild-type and *cat-2* speed traces with comparable average speeds illustrates that the continuous track speeds of individual *cat-2* animals fluctuate more than those of wild-type animals (Figure 3C).

To quantify this difference, we determined the standard deviations of the intra-track speeds among *cat-2* tracks. Since the magnitude of speed fluctuations of wild-type animals varied by average speed, we first grouped animals by average speed and then compared the speed fluctuations of animals moving at the same average speeds. While comparing the speed fluctuations of an entire group of animals could be biased by the average speed of the group, this method allowed us to compare strains with greater numbers of faster or slower moving animals without such bias. We found that the speed fluctuations of *cat-*2 tracks were much larger than those of wild-type tracks (Figure 4A). The average speeds of wild-type and *cat-2* animals did not change significantly over the assay period, precluding the possibility that the greater standard deviation of speeds for *cat-2* mutant animals might be caused by a significant increase or decrease in average speed over the period of the assay (data not shown). Differences in speed fluctuations were seen with both our newly isolated allele and the canonical *cat-2(e1112)* allele (Figure 5B). Mutations in *tph-1* and *tdc-1*, which perturb the synthesis of serotonin and tyramine/octopamine, respectively, did not cause increased fluctuations in speed (Data not shown). These data demonstrate that DA is required to restrict the range of speeds at which an individual animal travels. In other words, DA is required for the precision with which a normal animal maintains its speed.

### Dopamine-deficient mutants make unusually large adjustments to their speed

We next examined wild-type and *cat-2* acceleration and found that *cat-2* mutants exhibited greater total acceleration than did wild-type animals (Figure 4B). This greater total acceleration was a consequence of the fact that *cat-2* mutants achieve greater peak acceleration and accelerate for slightly longer periods of time (Figure 4CD). Thus, DA functions to restrict the magnitude of the accelerations and decelerations made by an individual, which explains why dopamine-deficient *cat-2* individuals exhibited a greater range of locomotion rates than individual wild-type animals.

To determine whether *cat-2* mutants exhibited greater speed fluctuations on bacteria because they exhibit a higher average speed, we suppressed the average speed of *cat-2* mutants with a second mutation. Mutation of the serotonin reuptake transporter gene *mod-5* greatly reduces the average speed of animals on bacteria [24] and suppressed the abnormally high locomotion rate of *cat-2* mutants on bacteria (Figure 5A). Compared to those of wild-type animals, the speed fluctuations of *mod-5(n3314)* mutants were not strongly reduced, and mutations in *mod-5* did not suppress the large fluctuations in speed associated with reduced DA signaling in *cat-2* mutants (Figure 5B). That *mod-5* suppressed the *cat-2* increased speed on bacteria without also suppressing the increased speed fluctuations demonstrates that speed on bacteria and fluctuations in speed are aspects of *C. elegans* behavior that are independently modulated by DA signaling.

### Dopamine modulates locomotion through the metabotropic D2-like dopamine receptor DOP-3 and G_αo_ protein GOA-1

Mutations in the metabotropic D2-like DA receptor *dop-3* and the G**_α_**_o_ protein *goa-1* cause locomotor hyperactivity and resistance to immobilization by exogenous DA [16], [21]. Mutation of *dop-3* can also suppress swimming paralysis induced by mutations in the *C. elegans* dopamine transporter *dat-1* [25]. We found that like *cat-2* mutants both *dop-3* and *goa-1* mutants also made unusually large adjustments to their speed and exhibited a greater range of instantaneous speeds (Figure 6A–C). The *dop-3(vs106)* strain we tested was backcrossed four times, and X-linked double mutants with *vs106* were created following the mutation by PCR. All *vs106*-containing strains retained the phenotype of abnormally high speed fluctuations, indicating that this phenotype is likely caused by the *vs106* mutation. Mutations in genes that encode other G protein-coupled biogenic amine receptors – *dop-1(vs100)*, *dop-2(vs105)*, *dop-4(ok1321)*, *ser-1(ok345)*, *ser-2(pk1357)*, *ser-4(ok512)*, *tyra-3(ok325)*, *F14D12.6(ok371)*, and *T02E9.3(ok568)* [21], [26], [27], [28], [29] – did not cause similar defects (data not shown).

Mutation of the D1-like DA receptor *dop-1(vs100)* suppresses the defect in food-dependent slowing associated with the *dop-3(vs106)* mutation [21]. By contrast, we found that the *dop-1(vs100)* mutation did not suppress the increased fluctuations in the speed associated with the *dop-3(vs106)* mutation (Figure 7A).

### Treatment with exogenous dopamine restores normal locomotion to mutants defective in dopamine synthesis

Treatment of *cat-2* mutant animals with exogenous DA restored their ability to make small adjustments to their speed and to restrict the range of speeds at which they travel (Figure 6D–F). Thus, *cat-2* mutants retain the ability to receive and transduce DA signals, indicating that reduced levels of DA in the adult worm are responsible for the behavioral defects associated with *cat-2* mutation. That exogenous DA rescued the excess speed fluctuations of *cat-2* mutants suggests that DA acts humorally in maintaining a precise speed. Exogenous DA did not rescue these defects in *dop-3* and *goa-1* mutants (Figure 6D–F). This finding is consistent with the proposed roles of these genes in the postsynaptic reception and transduction of DA signals, respectively (Figure 6D–F) [21], although *goa-1* also functions in other signaling pathways [30], [31] and does not only act directly downstream of *dop-3* in modulating speed.

### A wild-type copy of the *dop-3* gene can restore normal locomotion to *dop-3(vs106)* mutants

A transgene containing a genomic copy of the *dop-3* gene suppressed the large speed fluctuations of *dop-3(vs106)* mutants (Figure 7B). A *dop-3* cDNA construct with identical upstream and downstream sequences did not have rescuing activity, indicating that a full genomic copy of *dop-3* is necessary to rescue *dop-3(vs106)* defects and that sequences outside of the *dop-3* open reading frame do not have rescuing activity (Figure 7B). Previous studies concluded that *dop-3* is expressed in the GABAergic and cholinergic motor neurons of the ventral cord as well as in head and tail neurons [21]. Additional studies concluded that *unc-47* is expressed only in GABAergic neurons [32], *unc-17* is expressed only in cholinergic neurons [33], *tax-4* is expressed in a subset of sensory neurons [34], and *gcy-5* is expressed in a single sensory neuron [35]. We replaced the *dop-3* promoter sequences in our genomic construct with *unc-17*, *unc-47*, *tax-4*, and *gcy-5* promoter sequences in four separate constructs and found that all four suppressed the large speed fluctuations of *dop-3(vs106)* mutant animals. The *unc-17* heterologous promoter construct and *dop-3* genomic construct were both able to suppress speed fluctuations to below wild-type levels (Figure 7B).

These observations indicate that the full genomic open reading frame, including introns and exons, are necessary and sufficient to confer rescuing activity to our DNA constructs with all of the promoter constructs we tested.

## Discussion

Using an automated locomotion tracking system we discovered that DA is required to make small adjustments to speed to maintain a constant rate of locomotion. The D2-like DA receptor DOP-3 and the G**_α_**_o_ protein GOA-1 were essential for the reception and transduction of a DA signal that acts this processes. Treatment of dopamine-deficient mutants with exogenous DA completely restored the ability to make small adjustments to speed. *mod-5* mutations modulated food-dependent slowing but did not modulate fluctuations in speed. While the D1-like DA receptor *dop-1* plays a role in food-dependent slowing [21], *dop-1* did not appear to function in maintaining precise rates of speed. Similarly, *dop-2*, *dop-4*, and a number of other biogenic amine receptors we tested did not function in maintaining precise rates of speed. These findings indicate that DA functions independently in food-dependent slowing and maintaining precise rates of locomotion.

### Dopamine might act in monitoring the animal's locomotor activity to precisely maintain a steady speed

The only dopaminergic neurons in *C. elegans* are the two ADE, two PDE and four CEP sensory neurons [7]. These neurons have been proposed to function in the mechanosensory detection of a bacterial lawn and in the proprioceptive detection of deflections in the body of the animal while it is moving [16], [36], [37]. The ADE and PDE neurons also appear important for transitions from swimming to crawling behavior, a process that appears dependent on mechanosensation [20]. We suggest that the PDE neurons, the processes of which run the length of the animal, might become stimulated and release DA when the animal is moving.

Since exogeneous DA restored the ability of *dop-3* mutants to make normal adjustments to their speed, we conclude that the amount of DA released does not signal the speed of the animal. Rather we propose that DA release increases the efficiency by which animals integrate information about their current speed. Efficient monitoring of current speed would allow animals to maintain a more precise rate of locomotion specifically when the animal is moving.

Impaired detection of speed would lead to a reduced ability to detect movement that is too rapid or too slow, resulting in greater variability in both average speeds and instantaneous speeds. Such a deficit would also result in large acceleration and deceleration events, since an acceleration or deceleration event would terminate only when the animal has accelerated or slowed to an acceptable speed, the detection of which would be impaired.

### Dopamine is required for normal levels of locomotor activity in both *C. elegans* and humans

We found that in comparison to wild-type *C. elegans*, dopamine-deficient mutants more frequently exhibited periods of both low and high rates of locomotion and also had a reduced ability to make small adjustments to speed. Parkinson's disease is similarly characterized by seemingly opposite symptoms that include extended periods of abnormally high (rigidity) and low (bradykinesia) motor activity; in addition, Parkinson's patients have a reduced ability to make fine adjustments to movements (uneven gait, postural instability) [38].

We determined that the metabotropic D2 DA receptor homologue DOP-3 was required for the fine coordination of *C. elegans* locomotion. In humans, metabotropic DA receptors are the targets of agonists (bromocriptine, apomorphine, pramipexole, ropinirole, rotigotine) that constitute a family of Parkinson's therapies [39], [40]. In addition, administration of the DA precursor L-DOPA (levodopa) is the most effective treatment for Parkinson's symptoms [41], [42]. We discovered that pre-incubation of animals with exogenous DA restored normal locomotion to *C. elegans* mutants with reduced DA levels. Based on these data and our belief that pre-incubation with DA results in high levels of the neurotransmitter throughout the animal, we conclude that acute DA release is not required to restore normal locomotion to dopamine-deficient mutants.

We have shown that impaired DA signaling can cause a range of defects in locomotor coordination by *C. elegans*. We note that like *C. elegans cat-2* mutants, human patients with Parkinson's disease display a constellation of movement disorders that include both greatly reduced and abnormally high levels of motor activity as well as impaired motor coordination and that these clinical features can be rescued by treatment with the DA precursor L-DOPA [43], [44]. We suggest that perhaps some aspects of Parkinson's disease involve mechanisms similar to those responsible for the behavioral abnormalities of *cat-2* mutants. Although the anatomical dopaminergic circuitry in humans and worms is quite different, it is possible that further studies of dopamine-dependent movement disorders of *C. elegans* might identify molecular and cellular mechanisms that are disrupted in humans with Parkinson's disease.

## Materials and Methods

### Locomotion Assay

Locomotion assays were conducted according to Sawin et al. [16], except that in most cases, locomotion was recorded by an automated locomotion tracking system. Assay plates containing bacteria were made by spreading a drop of HB101 *E. coli* into the shape of a ring using the smooth bottom of a small glass test tube. Assay plates were incubated at 37°C overnight; only those containing a textured non-glassy-appearing bacterial lawn were used for locomotion assays. Assays were performed in a temperature-controlled room at 22°C. Assays began 5 min after animals were transferred to assay plates and lasted for 2 min. All experiments were performed on multiple days. Data were pooled for evaluation. Data for a wild-type control were collected on each assay day. A modified assay protocol was used to assay transgenic strains. In this protocol, well-fed animals were transferred to assay plates, and their movements were recorded for several 2 min intervals up to 12 min post-transfer.

### Automated Tracking System

We used a Sony XCD-X710 firewire camera at 1024×768 resolution to capture high-contrast dark-field images of our assay plates illuminated from the side by a ring of LEDs. Images were acquired at 1 Hz. The centers of mass (centroids) of all worms were found, and individual position recordings were assembled into tracks from centroid positions using the methods outlined by Clark et al. [45]. Animal speed was calculated by dividing the path distance an animal traveled by the time it took to travel that path. Each speed recording was calculated by averaging 5 speed measurements observed during a 5 sec sliding window to minimize the noise in centroid displacements. Such instantaneous speed measurements varied from 0 to roughly 0.3 mm per sec. Instantaneous acceleration rates were determined from these speed measurements.

The automated tracking software we used in our data capture and analysis is very similar to other freely available on-line packages that are more robust, inter-operable, better supported and easier to use than ours (MWT, MAGAT). A copy can be downloaded from GITHUB [46].

### Data Analysis

The average speed of a population was defined as the mean of all instantaneous speeds calculated for all animals tested.

To analyze the variation in speed recordings within individual tracks, we used only tracks that contained at least 30 seconds of recorded data. Tracks were first separated into bins by average speed. The standard deviation of the intra-track speed recordings was determined for each track and the average standard deviation for each bin was determined from these calculations.

To analyze average total acceleration, acceleration durations, and peak accelerations within individual tracks, we used only tracks that contained at least 30 seconds of recorded data. Tracks were first separated into bins by average speed. Total acceleration was calculated as the root mean square (RMS) of accelerations. The duration of an acceleration event was calculated as the time elapsed between when an animal's acceleration became greater/less than zero and then returned to zero. Peak acceleration/deceleration is the highest/lowest acceleration value of each acceleration/deceleration event. Average properties were calculated for each track. The average value for each bin was then determined from these calculations.

In the above analyses, data for each track (standard deviation of speed, RMS of acceleration, duration of acceleration and peak acceleration) were treated as an independent measurement for computing the means and SEMs shown in the graphs. Significance between genotypes was computed using a 2-way ANOVA. Significance between pairs of points within each bin or between average speeds was computed with a Student's t-test.

#### Strains

*Caenorhabditis elegans* strains were cultivated on NGM agar at 20°C as described by Brenner [13], except that worms were grown on *E. coli* strain HB101 as a food source. Other strains used in this paper are: AQ866 *ser-4(ok521)*, CB1112 *cat-2(e1112)*, DA1814 *ser-1(ok345)*, LX645 *dop-1(vs100)*, LX705 *dop-1(vs100) dop-3(vs106)*, LX702 *dop-2(vs105)*, LX703 *dop-3(vs106)*, MT2426 *goa-1(n1134)*, MT19600 *dop-3(vs106) lin-15AB(n765)*, MT9772 *mod-5(n3314)*, MT10661 *tdc-1(n3420)*, MT13710 *mod-5(n3314); cat-2(e1112)*, MT14984 *tph-1(n4622)*, MT15620 *cat-2(n4547)*, OH313 *ser-2(pk1357)*, RB785 *T02E9.3(ok568)*, RB1254 *dop-4(ok1321)*, VC125 *tyra-3(ok325)*, VC224 *F14D12.6(ok371)*. MT15620 *cat-2(n4547)* is a newly isolated strain that contains a 1010 bp deletion that removes sequences 3′ to ATGTGAAGTCACACCTGTCT and 5′ to ATCATTTTGAAAATCCGACC of the *cat-2* locus (Niels Ringstad, personal communication).

### Transgenic Strain Construction

The *dop-3* cDNA construct contained an RT-PCR-amplified cDNA fragment, 10 kb of upstream regulatory sequences that included the beginning sequence TAAACTTGGTTGACCCACCCC, and 1.5 kb of downstream sequences that included the ending sequence CCAGACGAGACGCTGAGATTGA. The transcriptional start site of the *dop-3* gene was determined by 5′ RACE and confirmed by SL1-primed isolation of the *dop-3* cDNA. The sequence of the *dop-3* cDNA construct was determined and corresponded to sequences that encode the DOP-3 protein (WP:CE39178) annotated in WormBase. The *dop-3*-rescuing construct contained the genomic sequences between and including the two sequences annotated above.

Four additional constructs were created by replacing *dop-3* promoter sequences in the genomic rescuing construct with heterologous promoter sequences. The *tax-4* promoter construct contained 5.3 kb of genomic DNA that was amplified with the primers CACACAACGGACAGTAATGGCG and TCTTGAAACATAATTAAATTTTGAGAATGATAGAAG. The *gcy-5* promoter construct contained 1.9 kb of genomic DNA that was amplified with the primers TTTCACAGTTGGCTCCACCTCTGG and TTTTCATCAGAATAAGTAATTTTTCGAAAAC. The *unc-17* promoter construct contained 4.2 kb of genomic DNA that was amplified with the primers GTTTTTGGGATTTTTGCGGG and CTCTCTCTCTCCCCCTGGAATATTTTAT. The *unc-47* promoter construct contained 2.9 kb of genomic DNA that was amplified with the primers AATGGGAGAATAAATGGGACGG and CTGTAATGAAATAAATGTGACGCTGTCG. Constructs were injected at 100 ng/µL into a *dop-3(vs106)* strain with a *myo-2::rfp* co-injection marker and into a *dop-3(vs106) lin-15(n765)* strain with a wild-type copy of *lin-15*. Both groups of transgenic animals yielded identical results; only data for *lin-15* rescued transgenic strains are shown in Figure 7.

### Drug Pretreatment

A fresh solution of 50 mM DA hydrochloride (Sigma) in M9 buffer [13] was prepared, and 400 µl of this solution was immediately added to a standard plate containing approximately 10 ml NGM agar seeded with a bacterial lawn. Plates were allowed to dry for 1 hr. Control plates were made by adding 400 µl M9 to standard plates containing approximately 10 ml NGM agar seeded with a bacterial lawn. Animals were incubated on dopamine-containing plates or control plates for 4–6 hr prior to assay. Assay plates and food-deprivation plates did not contain dopamine.
